# EFR-Mediated Innate Immune Response in *Arabidopsis thaliana* is a Useful Tool for Identification of Novel ERQC Modulators

**DOI:** 10.3390/genes10010015

**Published:** 2018-12-27

**Authors:** Andrea Lia, Antonia Gallo, Lucia Marti, Pietro Roversi, Angelo Santino

**Affiliations:** 1 Institute of Sciences of Food Production, C.N.R. Unit of Lecce, via Monteroni, I-73100 Lecce, Italy; andrea.lia@ispa.cnr.it (A.L.); antonia.gallo@ispa.cnr.it (A.G.); lucia.marti@uniroma1.it (L.M.); 2 Department of Biology and Biotechnology “C. Darwin”, Sapienza University of Rome, 00185 Rome, Italy; 3 Leicester Institute of Structural and Chemical Biology, Department of Molecular and Cell Biology, University of Leicester, Henry Wellcome Building, Lancaster Road, Leicester LE1 7RH, UK

**Keywords:** *Arabidopsis thaliana*, ER α–glucosidase II, glycoprotein folding quality control, UGGT, elf18, flg22, EFR, *rsw3*, *uggt1-1*, *N*B-DNJ.

## Abstract

Plants offer a simpler and cheaper alternative to mammalian animal models for the study of endoplasmic reticulum glycoprotein folding quality control (ERQC). In particular, the *Arabidopsis thaliana* (*At*) innate immune response to bacterial peptides provides an easy means of assaying ERQC function in vivo. A number of mutants that are useful to study ERQC *in planta* have been described in the literature, but only for a subset of these mutants the innate immune response to bacterial elicitors has been measured beyond monitoring plant weight and some physio-pathological parameters related to the plant immune response. In order to probe deeper into the role of ERQC in the plant immune response, we monitored expression levels of the Phosphate-induced 1 (*PHI-1)* and reticulin-oxidase homologue (*RET-OX)* genes in the *At* ER α-Glu II *rsw3* and the *At* UGGT *uggt1-1* mutant plants, in response to bacterial peptides elf18 and flg22. The elf18 response was impaired in the *rsw3* but not completely abrogated in the *uggt1-1* mutant plants, raising the possibility that the latter enzyme is partly dispensable for EF-Tu receptor (EFR) signaling. In the *rsw3* mutant, seedling growth was impaired only by concomitant application of the *At* ER α-Glu II *N*B-DNJ inhibitor at concentrations above 500 nM, compatibly with residual activity in this mutant. The study highlights the need for extending plant innate immune response studies to assays sampling EFR signaling at the molecular level.

## 1. Introduction

Glycoproteins traversing the secretory pathway of eukaryotic cells reach their cellular or extracellular destinations after folding in the endoplasmic reticulum (ER) [1]. Glycoprotein folding in the ER is aided by the ER glycoprotein folding quality control (ERQC) machinery, also known as the calnexin cycle [2]. A number of chaperones and foldases associate with the ER lectins calnexin (CNX) and/or calreticulin (CRT), which bind glycoproteins carrying an *N*-linked mono-glucosylated glycan, Glc_1_Man_9_GlcNAc_2_. ER α–glucosidase II (Glu II), the ERQC usher, mediates entry of a glycoprotein into the calnexin cycle by cleaving the outer glucose from a glycoprotein’s Glc_2_Man_9_GlcNAc_2_ *N*-linked glycan, yielding a mono-glucosylated *N*-linked glycan [3]: thanks to the action of Glu II on the native glycan, all glycoproteins have at least one chance to associate with CNX/CRT and profit from chaperone/foldase assisted folding.

Remarkably, the same ERQC usher allowing glycoprotein entry into the calnexin cycle, enables escape from it: after having mediated the exposure of a mono-glucosylated glycan, Glu II catalyzes a second Glc cleavage, removing the inner glucose from the glycan, thus making it no longer a substrate for ER lectins CNX/CRT. As every *N*-glycosylated glycoprotein originally carries one or more *N*-linked glycans, all glycoproteins can profit from one or more rounds of CNX/CRT association via the Glc residues present on the glycans upon entry into the ER. Indeed, simply thanks to the mono-glucosylated *N*-linked glycans generated by Glu II, easy/quick-to-fold glycoproteins bind ERQC lectins and chaperones/foldases long enough to form successfully, and then progress towards the Golgi down the secretory pathway.

Glycoproteins that are slower/trickier to fold need further rounds of interaction with ERQC lectins and chaperones/foldases. The ERQC checkpoint, UDP-glucose glycoprotein glucosyltransferase (UGGT), evolved to take care of these glycoproteins, preventing their premature exit from the ER. The enzyme recognizes a mis-folded glycoprotein and adds back a glucose residue to a Man_9_GlcNAc_2_ *N*-linked glycan. At this end of the cycle, many glycoproteins undergo multiple re-glucosylation and CRT/CNX binding events. UGGT deletion often results in premature release of these glycoproteins from the cycle. For other proteins, UGGT deletion substantially delays release from calnexin [4], perhaps because UGGT itself associates with chaperones required for structural maturation export from the ER [5].

Confirming the importance of Glu II and UGGT in development, no multicellular organism is known to survive Glu II KO to adulthood [6], and homozygous UGGT deletion is embryonically lethal in mice [7]. Mutations to either the Glu II or UGGT genes can impair glycoprotein folding and cause ER retention and/or degradation, with the accompanying loss of function [8,9,10,11]. Yet, the centrality of ERQC to viral glycoprotein folding and secretion makes Glu II and UGGT appealing targets for host-acting antivirals, with clinical relevance for broad-spectrum antiviral therapy. Indeed, iminosugars (a class of glycomimetics) are antiviral ER α-Glu II inhibitors that are well tolerated in mammals [12], with ER α-Glu iminosugar inhibitors having reached clinical trials against dengue fever (https://clinicaltrials.gov/ct2/show/NCT01619969) and HIV (in combination with AZT, [13]). UGGT too has been advocated as a pharmacological target, UGGT modulation having therapeutic potential for the rescue of the secretion of misfolded but functional glycoprotein mutants in congenital protein folding diseases [14,15,16]. No UGGT inhibitors are known (apart from its product, UDP [17]); heterozygous UGGT_1_^+/−^ knockout mice have been reported to express approximately half of the wild-type (WT) amount of UGGT_1_ but they undergo normal development and have no obvious aberrant phenotype [18].

The high conservation of sequence and function of ERQC components across eukaryotes makes it possible to use fungi and plants for basic studies of ERQC in cellula [19] and in vivo [20], respectively, without the ethical and economical complications of mammalian animal models. Any phenotype mediated by glycoproteins whose folding is controlled by ERQC enables the study of ERQC function in vivo. In plants, such phenotypes are provided by the response to microbe-associated molecular patterns (MAMPs), measured in terms of MAMP-induced events, e.g., reduced plant weight or detectable reactive oxygen species (ROS) generation [9].

Amongst the best studied MAMPs are bacterial proteins such as EF-Tu (elongation factor thermo unstable), recognized by the EF-Tu receptor (EFR), a pattern-recognition receptor in *Arabidopsis thaliana* (*At*) [21]. Plant cells recognize and bind EF-Tu, preventing genetic transformation and protein synthesis in pathogens such as *Agrobacterium tumefaciens* [21]. *At* Glu II point mutations and deletions impair the plant response to bacterial peptide elf18, derived from EF-Tu, likely because the EF-Tu receptor (EFR) or another protein in the elf18 signaling pathway requires calnexin cycle assisted folding [21]. Two more genetic studies showed that UGGT—like Glu II—is also important for the correct function of the plant innate immune response to elf18 [8,9]. The implication of those studies is that EFR and/or a glycoprotein involved in elf18 EFR-mediated signaling depend on repeated cycles of association with the ER lectins and the associated chaperones and foldases—explaining the need for both ER α-Glu II and UGGT.

Somewhat surprisingly, the plant immune response to a different bacterial peptide, flg22, mediated by the flagellin sensing 2 receptor (FLS2), which belongs to the same fold family as the EFR receptor (subfamily XII of the leucine-rich repeat receptor kinases also known as LRR-RKs), is not affected by Glu II impairment [22] nor by one of the UGGT mutants that impair elf18 response [9]. Loss of *At* UGGT also shows no discernable defects in the plant response to the brassinosteroid hormone, mediated by the BRI1 receptor, another member of the LRR-RK family.

A number of mutants that are useful to study ERQC in planta have been described in the literature, but for most of them the innate immune response to bacterial elicitors has been measured only by monitoring plant weight and some physio-pathological parameters related to plant immune response. In order to probe deeper into the role of ERQC in the plant immune response, we monitored expression levels of the Phosphate-induced 1 (*PHI-1)* and reticulin-oxidase homologue (*RET-OX)* genes encoding respectively for phosphate-induced 1 and reticuline oxidase, in the *At* ER α-Glu II *rsw3* and the *At* UGGT *uggt1-1* mutant plants, in response to bacterial peptides elf18 and flg22. The elf18 response was impaired in the *rsw3* but not completely in the *uggt1-1* mutant plants. Moreover, in the *rsw3* mutant, seedling growth was impaired only by concomitant application of the *At* ER α-Glu II *N*B-DNJ inhibitor at concentrations above 500 nM, suggesting residual activity of the *At* ER α-Glu II *rsw3* mutant and/or extra toxic effects due to *N*B-DNJ glycolipid metabolism inhibition [23]. The study highlights the need for extending plant innate immune response studies assays to assays sampling ERQC function at the molecular level.

## 2. Materials and Methods

### 2.1. Plant Material, Growth Conditions, and Treatments

#### 2.1.1. Testing the Effect of Elf18/Flg22 Elicitors and *N*B-DNJ on Plant Weight

The surface of the seeds was sterilized, the seeds were vernalized for 4 days prior being sown in 12-wells plates containing one-half strength Murashige and Skoog (MS) medium [24]. About 10 seeds were placed per each well containing 2 mL of liquid medium supplemented with 0.5% (*w*/*v*) sucrose. 70 μM *N*B-DNJ treatment was carried out on three-day-old seedlings germinated and grown in one-half-strength MS medium, together with 100 nM elf18 (ac-SKEKFERTKPHVNVGTIG) or flg22 (QRLSTGSRINSAKDDAAGLQIA) peptide elicitors. The effect of treatment on seedling growth was analyzed 13 days post sowing by photography and recording the fresh weight of plants. To test the toxicity of *N*B-DNJ on *rsw3* plants *N*B-DNJ was used in different concentrations (10 µM, 5 µM, 2 µM, 1 µM, 500 nM, 300 nM, 100 nM, 50nM) on three-day-old seedlings. Seedlings were grown at 22°C and 70% relative humidity under a 16-h-light/8-h-dark cycle (approximately 120 µmol m^–2^ s^–1^). Statistical analysis of the weights was performed using the software GraphPad-Prism v5.03 (La Jolla, USA), test ANOVA with Bonferroni’s test.

#### 2.1.2. Growth Condition for Gene Expression Analysis Experiment

**Figure 1:**
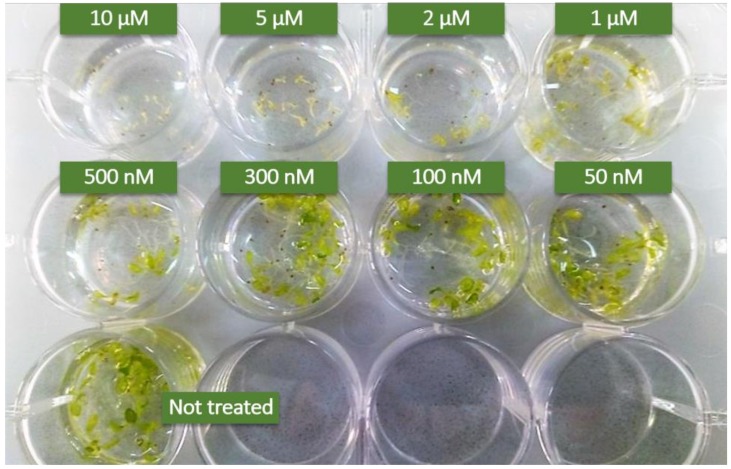
Effects of *N*B-DNJ-treatment on the *Arabidopsis thaliana rsw3* mutant. 15 days-old *rsw3* plants grown with different concentration of *N*B-DNJ. Each well contains about 10 seeds. Above 500 nM, *N*B-DNJ impairs plant embryo growth. Results derive from three independent experiments (± s.e. *n* = 10).

**Figure 2:**
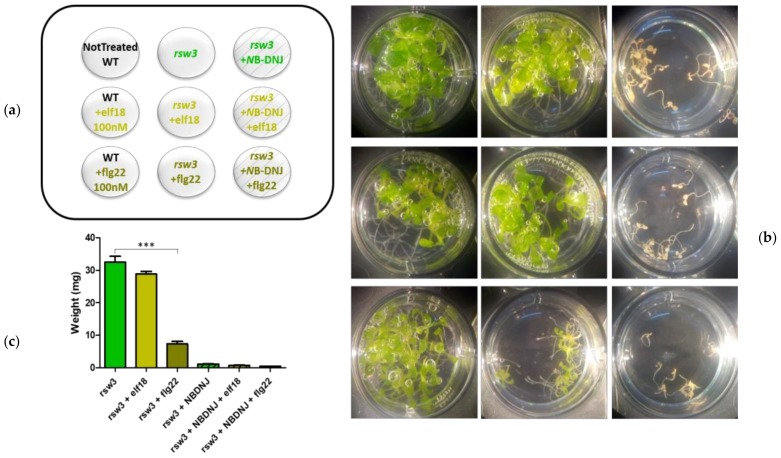
Effects of elicitors-treatment on *rsw3* mutant. 13-day-old seedlings control or treated with 100 nM elf18 or flg22 elicitors with and without 70 µM *N*B-DNJ. (**a**) Scheme of the treatments distribution on the plate. The nine wells are depicted, each with the plant genotype and the amounts of *N*B-DNJ and/or elf18/flg22 used. (**b**) Images of the plants after the treatments. Each well contains about 10 seedlings. (**c**) Mean of the plants fresh-weight (expressed in mg) of at least three independent experiments (± s.e. *n* = 10), statistical analysis determined by ANOVA with Bonferroni’s test (* = *p* < 0.5, ** = *p* < 0.01, *** = *p* < 0.001). WT: wild type.

For gene expression analysis the surface of the seeds was sterilized, they were vernalized for four days and sown in multiwell plates (approximately 10 seeds per well) containing one-half strength MS medium with 0.5% sucrose (*w*/*v*) (2 mL per well). After 5 days 70 μM of *N*B-DNJ was added to the medium in the wells as indicated in Figure 1A, Figure 2A and Figure 4A. After 9 days, the medium was replaced with fresh medium (plus *N*B-DNJ as indicated) and treatments with elicitors elf18 and flg22 (100 nM) were performed after 24 h (the 10th day) for 30 min before freezing the tissues in liquid nitrogen.

### 2.2. Gene Expression Analysis

After treatment with elicitors, 10-day-old seedlings were frozen and ground with pestle and mortar in liquid nitrogen. Total RNA was extracted from three independent replicates, each composed of 20 seedlings, using the NORGEN-Total RNA Purification Kit (Biotek Corp., Thorold, ON, Canada) according to the manufacturer’s protocol. RNA samples were treated with RNase-free DNase I (ThermoFisher scientific, Waltham, MA, USA) to eliminate any potential DNA contamination. First-strand cDNA was synthesized from 1 µg of total RNA by using Maxima H Minus cDNA Synthesis Master Mix (ThermoFisher scientific) according to the manufacturer’s instructions. Quantitative reverse transcription-PCR (qRT-PCR) was used to analyze the expression level of the *PHI1* and *RET-OX* genes [25]; the constitutively expressed *UBIQUITIN5* gene served as an internal reference to normalize gene expression. Analysis was performed on a StepOne™ Real-Time PCR System (Applied Biosystem, Warrington, United Kingdom) in a reaction volume of 20 µL containing 2X iTaq Universal SYBR Green Supermix (Bio-Rad, Hercules, CA, USA) and 0.5 µM of each primer. The following amplification conditions were used: an initial denaturation step at 95 °C for 2 min, followed by 40 cycles of 15 s at 95 °C and 1 min at 60 °C. Specificity of the PCR amplification was confirmed by dissociation curve analyses. The relative quantification of gene expression was established using the comparative 2^−ΔΔCT^ method. The PCR efficiency of each oligonucleotide pair was calculated from linear regressions of the standard curves. Real-time PCR derived data were relatively quantified taking into account the divergent efficiencies [25]. Three replicates were performed for each experiment in addition to a no-template control included for each primer pair. Statistical analysis was performed using Student’s *t*-test. Primers sequences are shown in Table S1.

### 2.3. Homology Modeling

Homology modeling of *A. thaliana* UGGT (Uniprot database entry UGGG_ARATH) was carried out using the HHPred server [26] to align the sequence of *At* UGGT with the one of the *Chaetomium thermophilum* orthologue and the homology model created with MODELER [27]. The figure was made with PyMol [28].

## 3. Results

### 3.1. Development of *A. thaliana* ER α-Glu II rsw3 Mutant Seedlings is Sensitive to NB-DNJ Iminosugar Dose Higher than or Equal to 500 nM.

Two missense mutants have been reported in *Arabidopsis* for the ER α–Glu II α catalytic subunit: the *At* Glu II S517F also known as *psl5*-1 mutant and the *At* ER α–Glu II S599F also known as *rsw3* mutant (Table S2). Both these mutants grow normally under standard conditions but show impaired response to elf18 treatment [29]. Both *psl5-1* and *rsw3* are missense mutations, raising the question as to any residual ER α–Glu II activity. In previous work, we showed that germinated embryos of *At psl5-1* were unable to growth in the presence of 70 µM *N*B-DNJ iminosugar [20], suggesting that *At psl5-1* may possess residual activity, the latter only completely abrogated by the iminosugar treatment. Alternatively, the extra toxic effects due to *N*B-DNJ glycolipid metabolism inhibition may explain the toxicity [23]. As the site of the *At* Glu II *rsw3* S599F amino acid substitution is located further away from the catalytic residues (*At* Glu II Asp512 and Asp588) than the site of the S517F mutations in *psl5-1* [20], it is expected that the *rsw3* mutant may also carry residual activity, as suspected also by Soussillane et al. [29].

To check if this is the case, we repeated the *N*B-DNJ iminosugar treatment on the *At* ER α–Glu II *rsw3* mutant. We then treated *rsw3* embryos with elf18 or flg22 elicitors. Concentrations of *N*B-DNJ lower 500 nM were not toxic to *rsw3* mutants: embryo growth was not significantly impaired by 300 nM *N*B-DNJ (Figure 1). At *N*B-DNJ iminosugar dose higher than or equal to 500 nM, the growth of *rsw3* embryos was completely abolished (Figure 1).

To check the ability of the *rsw3* plant to respond to bacterial peptides, we preliminary evaluated the response of three-days-old WT plants (Col-0 ecotype) to elf18 or flg22 elicitation (for 10 days). As expected, both treatments reduced significantly plant growth (Figure S1). In our hands, the *rsw3* mutant was insensitive to elf18 and highly sensitive to flg22 (Figure 2B,C), in keeping with what is reported in the literature [28].

### 3.2. *A. Thaliana* ER α-Glu II Rsw3 is INSENSITIVE to EFR Signaling

**Figure 3:**
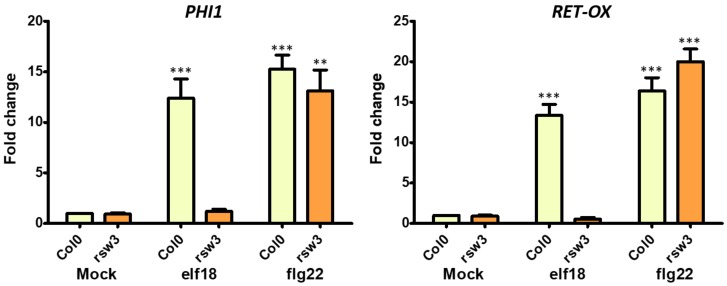
Expression of defence genes in the *At rsw3* plant. 10-day-old seedlings were treated with water or elf18 and flg22 elicitors. Expression of phosphate-induced 1 (*PHI-1*) and reticulin-oxidase homologue (*RET-OX*) genes were determined by quantitative reverse transcription-PCR (qRT-PCR) 30 min after treatment. Transcript levels are shown as the mean of at least three independent experiments (± s.e. *n* = 20 in each experiment) normalized to ubiquitin 5 (*UBQ5)* used as a reference. In both panels, asterisks indicate statistically significant differences among mock and treatments within the same genotype (same color) according to Student’s *t*-test (* = *p* < 0.5, ** = *p* < 0.01, *** = *p* < 0.001).

Next, we investigated the expression level of two genes typically induced by elf18 and flg22 elicitation. Phosphate-induced 1 and *RET-OX* are well known early elicitor-induced genes [30]. 10-day-old Col-0 seedlings and *rsw3* seedlings were treated with elf18 (or flg22 used as a control) for 30 min. Low expression levels of both *PHI-1* and *RET-OX* were recorded in the *rsw3* mutant seedlings in response to elf18 but not flg22, while Col0 seedlings showed a normal defense response characterized by higher expression levels of defense genes with both elicitors (Figure 3).

Furthermore, *rsw3* mutant plants were in our hands fully resistant to elf18 elicitation, as confirmed by plant weight and expression profiles of defense genes. For this reason, we did not further investigate in this mutant the effect of 0.5 µM iminosugar treatment on the expression levels of defense genes.

### 3.3. *A. Thaliana* UGGT uggt1-1 Mutant Retains Some EFR Signaling

At the calnexin cycle checkpoint end, both insertional UGGT mutants and point mutants were previously reported (Table S3). In the first category fall *uggt1-1* and *uggt1-2*, (carrying T-DNA insertions in the 11th and 16th exons, respectively [10]) and the *uggt-4* mutant, characterized by a T-DNA insertion in the 24th intron [8]. With regard to point mutants that showed impaired immune response to EFR, Saijo et al. [9] reported three point mutants (psl2-1: *At*UGGT D1497N, *psl2-2*: *At*UGGT E306K and *psl2-4*: *At*UGGT W1443STOP), together with a double mutant (*psl2-3*: *At*UGGT R1409K/W1443STOP) and a large deletion mutant (*psl2-5*: *At*UGGT ∆304-1613). Furthermore, Li et al. [8] reported another point mutant of UGGT, which was named uggt-3: *At*UGGT W1523STOP. We mapped these mutants on a homology model of *At*UGGT (SI, Figure S2).

Concerning the insertional mutants *uggt1-1* and *uggt1-2*, Blanco-Herrera et al. [10] reported some residual re-glucosylation activity for *uggt1-1* in comparison with *uggt1-2*, used in the same study. Both these mutants were reported to be more susceptible to pathogen attack, but their response to bacterial elicitors has not been investigated so far.

However, the *uggt1-1* mutant is characterized by a T-DNA insertion in the 11th exon, at a position corresponding to amino acid *At* UGGT H499, and thus much earlier than the *uggt 1-2* mutant (where T-DNA was mapped on the 24th intron) and the uggt4 mutant (where T-DNA was mapped on the 16th exon) for which efl18 insensitivity was reported [8]. We chose to investigate the *A. thaliana* T-DNA UGGT insertion mutant (*uggt1-1*) using EFR signaling. To confirm the dependency of the elf18 response on *At* ER α–Glu II we decided to investigate the effects of *N*B-DNJ treatment on the *uggt1-1* mutant response to elf18 elicitation, using the plant response to flg22 as a positive control.

**Figure 4:**
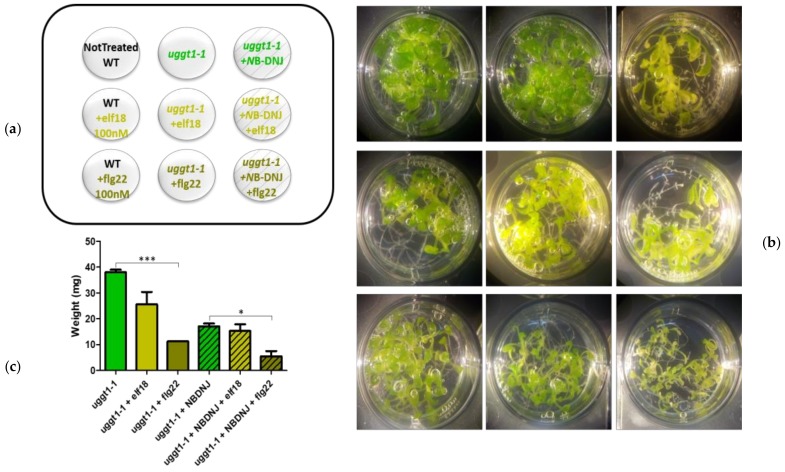
Effects of elicitor treatments on the *At uggt1-1* mutant. 13-day-old seedlings control or treated with 100 nM elf18 or flg22 elicitors with and without 70 µM *N*B-DNJ. (**a**) Scheme of the treatment distribution on the plate. The nine wells are depicted, each with the plant genotype and the amounts of *N*B-DNJ and/or elf18/flg22 used. (**b**) Images of the plants after the treatments. Each well contains about 10 seedlings. (**c**) Mean of the plants fresh-weight (expressed in mg) of at least three independent experiments (± s.e. *n* = 10), statistical analysis determined by ANOVA with Bonferroni’s test (* = *p* < 0.5, ** = *p* < 0.01, *** = *p* < 0.001).

Results shown in Figure 4 indicated that, unlike what was observed with the Glu II mutant *rsw3*, *N*B-DNJ is not lethal in the background of the *uggt1-1* mutant: treatment with the iminosugar caused a growth reduction similar to that observed in Col0 WT plants.

Elicitation experiments showed that this mutant was highly susceptible to flg22 (about 70% weight reduction). Elf18 elicitation impacted on *uggt1-1* plant growth much less than flg22, causing a lower plant weight reduction (about 35%), which was not statistically significant when compared to untreated plants. Only co-treatment of *At uggt1-1* with 70 µM *N*B-DNJ caused complete insensitivity to elf18, likely because of Glu II inhibition (Figure 4B,C).

**Figure 5:**
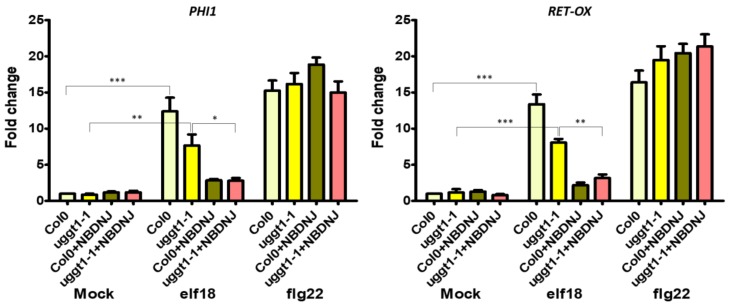
Expression of defence genes in the *At uggt1-1* plant. 10-day-old seedlings were treated with water or elf18 and flg22 elicitors, with and without 70 µM *N*B-DNJ. Expression of *PHI1* and *RET-OX* genes were determined by qRT-PCR 30 min after treatment. Transcript levels are shown as the mean of at least three independent experiments (± s.e. *n* = 20 in each experiment) normalized to *UBQ5* (ubiquitin 5) used as a reference. In both panels, asterisks indicate statistically significant differences among mock and treatments within the same genotype (same color) according to Student’s *t*-test (* = *p* < 0.5, ** = *p* < 0.01, *** = *p* < 0.001).

Finally, we verified the expression of innate immune defence genes in the *uggt1-1* mutant upon elf18 or flg22 elicitation. Under our experimental conditions, we found that both elicitors triggered a significant increase in the expression levels of both *PHI-1* and *RET-OX* and only the concomitant application of *N*B-DNJ blocked the EFR signaling pathway (Figure 5). *N*B-DNJ treatment induces resistance to elf18 in the same manner in WT and *uggt1-1* plants: indeed, there is no statistical difference between *PHI* and *RET-OX* transcript levels in WT and *uggt1-1* plants after treatment with elf18.

## 4. Conclusions

High sequence and functional conservation of the two main components of the ERQC, ER α–Glu II, and UGGT, enables the use of plant ER α–Glu II and UGGT mutants for in vivo ERQC studies. In a previous piece of work [16] we were able to recomplement the insertional UGGT mutant in the genetic background ebs1-3/bri1-9 using a functional UGGT from the thermophilic filamentous fungus *C. thermophilum*. In a separate study, we showed that the iminosugar *N*B-DNJ, which reached phase I clinical trials as an antiviral drug, was able to inhibit *A. thaliana* ER α–Glu II [20]. Taken together these results confirmed the potential of *A. thaliana* as a low-cost, easy-to-use organism to study ERQC function. In this context, leucine-rich repeat receptor kinases (LRR_RKs) are very useful ERQC client glycoproteins. Even though EFR, FLS2 and the brassinosteroid receptor BRI1 belong to the same sub-family (LRR-RK, sub-family XII), EFR-mediated signaling depends on ERQC, while the BRI1- and FLS2-mediated responses do not [8].

A possible explanation of this different behaviour is that EFR (or one of its downstream effectors) evolved more recently and depends on the ERQC machinery more than FLS2 and BRI1 [8]. Alternatively, macroscopic phenotypes such as loss of plant weight or decreased plant size are not subtle enough to probe the complex roles that ERQC components play in the folding of the many glycoproteins likely involved in the plant’s responses to elicitors. Differences in protein structure, and/or numbers of *N*-linked glycans and/or the relative positions of the *N*-glycosylation site(s) with respect to the sites of misfold can all affect the degree to which either the EFR/FLS2/BRI1 receptors, or other glycoproteins involved in their signaling cascades, depend on Glu II and UGGT.

Our results confirm those from Lu et al. [9], who showed for the first time that EFR-dependent ligand binding activity was impaired in *psl5-1* and *rsw3* mutants. While a T-DNA insertion mutant was reported for the β-subunit of ER α–Glu II [9], homozygous insertional mutants disrupting the fold of the α catalytic subunit of ER α–Glu II cause embryonic death [6], pointing to a likely essential role of the enzyme for seedling development. Interestingly, treatment of ER α–Glu II *rsw3* mutant with *N*B-DNJ at concentrations higher than 500 nM (see Figure 1) was lethal to *A. thaliana* embryos, likely because of complete inhibition of *At* Glu II, and/or extra toxic effects due to *N*B-DNJ glycolipid metabolism inhibition [23]. It is possible that the extra toxicity could be explained by inhibition of Glu I by *N*B-DNJ. However, concentrations of *N*B-DNJ higher than 100 µM are required to achieve Glu I inhibition in cellula [31]. To check for Glu I inhibition, tri-glucosylated glycans on glycoproteins would need quantifying with and without *N*B-DNJ treatment in the *rsw3* mutant.

Taken together, our results confirm that ER α–Glu II is essential for seedling development and EFR signaling, and that *At* Glu II *rsw3* may retain some residual activity.

In the *At uggt1-1* mutant, EFR signaling as measured by plant size is not significantly impaired, as reported in previous works for the *psl2-1*, *uggt-3*, and *uggt-4* UGGT mutants [9]. Yet, under our experimental conditions, both elicitors triggered a significant increase in the expression levels of both *PHI-1* and *RET-OX*, and only the concomitant application of *N*B-DNJ blocked completely the EFR signaling pathway (Figure 5). These results indicate that EFR-mediated *PHI-1* and *RET-OX* upregulation depends on glycoproteins that can fold successfully thanks to mono-glucosylated glycans afforded by ER α–Glu II alone and thus, UGGT activity is not needed for some of the EFR response. We note that both the WT and the *uggt 1-1* mutant survive 70 µM *N*B-DNJ treatment, which suggests that this dose of iminosugar only affords partial inhibition of *At* α–Glu II.

Finally, the observation that insertional mutants have been reported for *At* UGGT but not for ER α–Glu II indicate that UGGT activity modulation might have a less drastic impact on the glycoprotein folding machinery and ER homeostasis than ER α–Glu II deletion, boding well for future studies of UGGT modulation for the rescue of the secretion of misfolded but functional glycoprotein mutants in congenital protein folding diseases.

## Supplementary Materials

The following are available online at http://www.mdpi.com/2073-4425/10/1/15/s1, Figure S1: Effects of elicitors treatment on *At* Col0, Figure S2: Mapping of *At* UGGT mutants on a homology model, Table S1: Primers used in the qRT-PCR analysis showed in this work, Table S2: Known *At* ER α–Glu II mutants, Table S3: Known *At* UGGT mutants.
