# Supplementary material for: EFR-Mediated Innate Immune Response in Arabidopsis thaliana is a Useful Tool for Identification of Novel ERQC Modulators
Source: Genes (Basel). 2018 Dec 27;10(1):15. doi: 10.3390/genes10010015 (PMC6357087; doi:10.3390/genes10010015)
Supplement: Supplementary file 1 [file genes-10-00015-s001.pdf]

Supplementary information

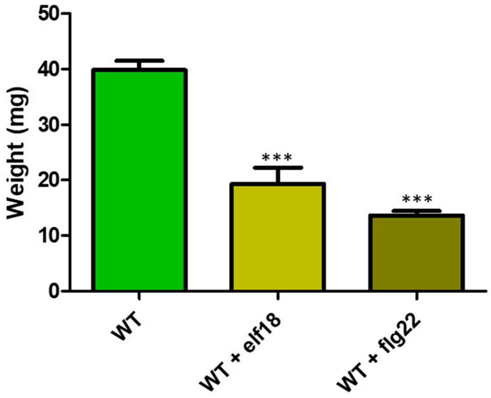

Supplementary Figure 1. Effects of elicitors treatment on *At* Col0. 13-days-old seedlings control or treated with 100 nM elf18 or flg22 elicitors. Values represent the mean of the plants fresh-weight (expressed in mg) of at least three independent experiments (± s.e. n=10). Statistical analysis was determined by ANOVA with Bonferroni's test (*=P<0.5, **=P<0.01, ***=P<0.001).

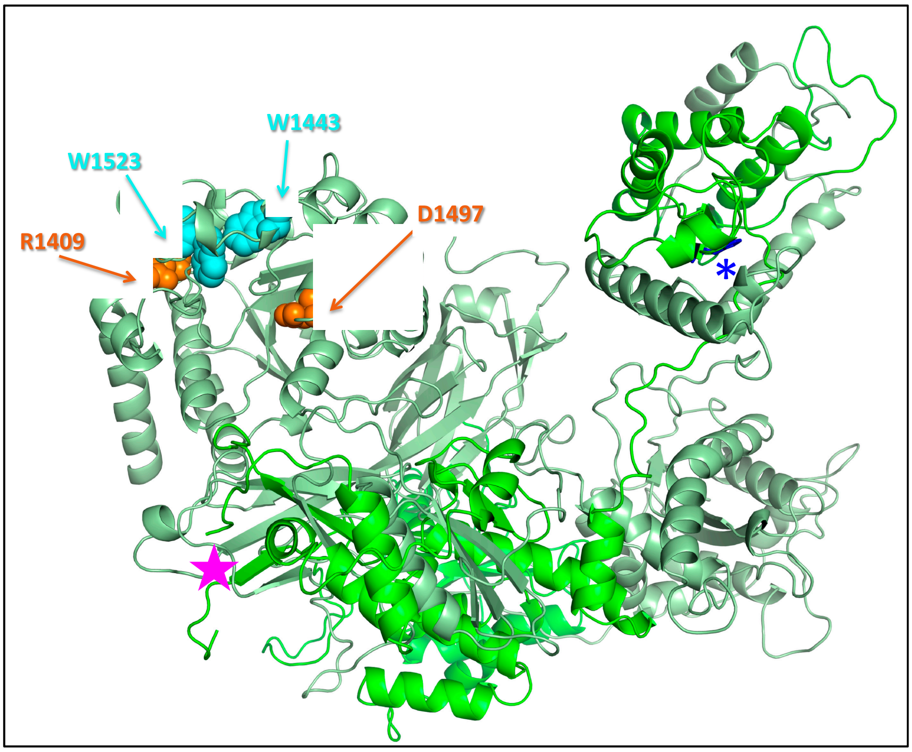

Supplementay Figure 2. Mapping of *At* UGGT mutants on a homology model. The portion of *At* UGGT preceding the site of the *psl2-5* Δ604-1613 deletion mutant is in bright green; in grey/green the portion of *At* UGGT that is missing in the same *psl2-5* mutant. In cyan spheres, the sites of the premature stop mutations (*uggt3:* W1523stop and *psl2-4:* W1443stop); in orange spheres, the missense mutants (*psl2-3:* R1409K and *psl2-1:* D1497N. The purple star marks the missing loop containing the site of the *psl2-2* E306K mutation. The blue asterisk marks the site of the T-DNA insertion in the *uggt1-1* mutant whose response to elicitors was characterised in this study.

Supplementary Table 1. Primers used in the qRT-PCR analysis showed in this work.

GENE	AG CODE	FORWARD PRIMER (5'-3')	REVERSE PRIMER (5'-3')
UBQ5	AT3G62250	GGAATCGACGCTTCATCTCG	ATGAAAGTCCCAGCTCCACA
PHI1	AT1G35140	TTGGTTTAGACGGGATGGTG	ACTCCAGTACAAGCCGATCC
RET-OX	AT1G26380	AGGTTCTCGAACCCTAACAACA	GCACAGACGACACGTAAGAAAG

Supplementary Table 2. Known *At* ER α–Glu II mutants.

Mutant name	Kind of mutation	Was it tested for elf18 response?	References
psl5-1	S517F	Yes	*[28]*
rsw3	S599F	Yes	
psl4-1	T-DNA insertion	Yes	
psl4-2	W360stop	Yes	

Supplementary Table 3. Known *At* UGGT mutants.

Name of mutant	Kind of mutation	Was it tested for elf18 response?	References
uggt1	Aberrant mRNA splicing variant	No	*[8]*
uggt2	Aberrant mRNA splicing variant	No	
uggt3	W1523stop	Yes	
uggt4	T-DNA insertion in 16th exon	Yes	
uggt1-1	T-DNA insertion in 11th exon	No	*[10]*
uggt1-2	T-DNA insertion in 24th intron	No	
psl2-1	D1497N	Yes	*[9]*
psl2-2	E306K	No	
psl2-3	R1409K, W1443stop	No	
psl2-4	W1443stop	No	
psl2-5	Δ604-1613	No	
